# Explicit Training to Improve Affective Prosody Recognition in Adults with Acute Right Hemisphere Stroke

**DOI:** 10.3390/brainsci11050667

**Published:** 2021-05-20

**Authors:** Alexandra Zezinka Durfee, Shannon M. Sheppard, Erin L. Meier, Lisa Bunker, Erjia Cui, Ciprian Crainiceanu, Argye E. Hillis

**Affiliations:** 1 Department of Neurology, Johns Hopkins University School of Medicine, Baltimore, MD 21205, USA; adurfee1@jhmi.edu (A.Z.D.); e.meier@northeastern.edu (E.L.M.); lbunker3@jhmi.edu (L.B.); 2 Department of Communication Sciences and Disorders, Chapman University, Irvine, CA 92618, USA; ssheppard@chapman.edu; 3 Department of Communication Sciences and Disorders, Northeastern University, Boston, MD 02115, USA; 4 Department of Biostatistics, Johns Hopkins University, Baltimore, MD 21205, USA; ecui1@jhmi.edu (E.C.); ccraini1@jhmi.edu (C.C.); 5 Department of Physical Medicine and Rehabilitation, Johns Hopkins University, Baltimore, MD 21287, USA; 6 Department of Cognitive Science, Johns Hopkins University, Baltimore, MD 21218, USA

**Keywords:** right hemisphere, stroke, prosody, emotion, affect, recognition, training

## Abstract

Difficulty recognizing affective prosody (receptive aprosodia) can occur following right hemisphere damage (RHD). Not all individuals spontaneously recover their ability to recognize affective prosody, warranting behavioral intervention. However, there is a dearth of evidence-based receptive aprosodia treatment research in this clinical population. The purpose of the current study was to investigate an explicit training protocol targeting affective prosody recognition in adults with RHD and receptive aprosodia. Eighteen adults with receptive aprosodia due to acute RHD completed affective prosody recognition before and after a short training session that targeted proposed underlying perceptual and conceptual processes. Behavioral impairment and lesion characteristics were investigated as possible influences on training effectiveness. Affective prosody recognition improved following training, and recognition accuracy was higher for pseudo- vs. real-word sentences. Perceptual deficits were associated with the most posterior infarcts, conceptual deficits were associated with frontal infarcts, and a combination of perceptual-conceptual deficits were related to temporoparietal and subcortical infarcts. Several right hemisphere ventral stream regions and pathways along with frontal and parietal hypoperfusion predicted training effectiveness. Explicit acoustic-prosodic-emotion training improves affective prosody recognition, but it may not be appropriate for everyone. Factors such as linguistic context and lesion location should be considered when planning prosody training.

## 1. Introduction

Affective (emotional) prosody refers to the suprasegmental aspects of speech, including pitch, volume, rate, and rhythm, that convey emotional meaning or intent [1,2]. Stroke-induced right hemisphere damage (RHD) can lead to impaired affective prosody recognition (termed receptive aprosodia) and expression (termed expressive aprosodia) and can thus cause significant communication breakdowns between these individuals and their loved ones, caregivers, colleagues, and fellow community members. Receptive aprosodia has been linked to reduced quality of life in various neurological conditions, such as stroke [3], dementia [4], and traumatic brain injury [5], as well as to poorer psychosocial functioning in psychological conditions, such as major depressive disorder [6]. Poor affect recognition has also been identified as a significant issue by caregivers of individuals with RHD [7]. Blonder and colleagues [8] observed that individuals with RHD who had poorer facial affect perception and non-affective linguistic prosody discrimination (i.e., determining if an utterance is a question or a statement based on intonation cues) had reduced satisfaction in their personal relationships. As can be gleaned from these examples, paralinguistic features are critical to convey communicative intent not inherent in the linguistic content of a message alone, and disruption of the encoding or decoding of these features can lead to pervasive interpersonal dysfunction.

In one of the earliest accounts of affective prosody processing following RHD, Ross [9] reported aprosodia profiles that mirrored the classic aphasia taxonomy following left hemisphere stroke. He identified a case of sensory aprosodia (Case 4) as demonstrated by intact production capability—albeit not always applied appropriately—and poor repetition and comprehension of affective prosody due to a posterior temporoparietal lesion, a roughly right-hemisphere homologue to Wernicke’s area. Recent models of propositional language organization have moved from discussing functional localization in terms of neural substrate “islands” (with damage to cortical regions resulting in mutually exclusive aphasia subtypes) to models comprising these cortical regions as well as their connections via white matter pathways, representing ventral and dorsal processing streams [10,11,12]. A similar topography is observed in the right hemisphere for affective prosody, wherein more dorsally situated structures are implicated in production and more ventrally located regions in recognition [13,14,15]. Focusing specifically on affective prosody recognition, right hemisphere temporoparietal regions [12,16,17,18,19,20,21,22] as well as frontal [2,14], subcortical [15,21,22,23], and white matter [24] structures have been implicated.

**Figure 1:**
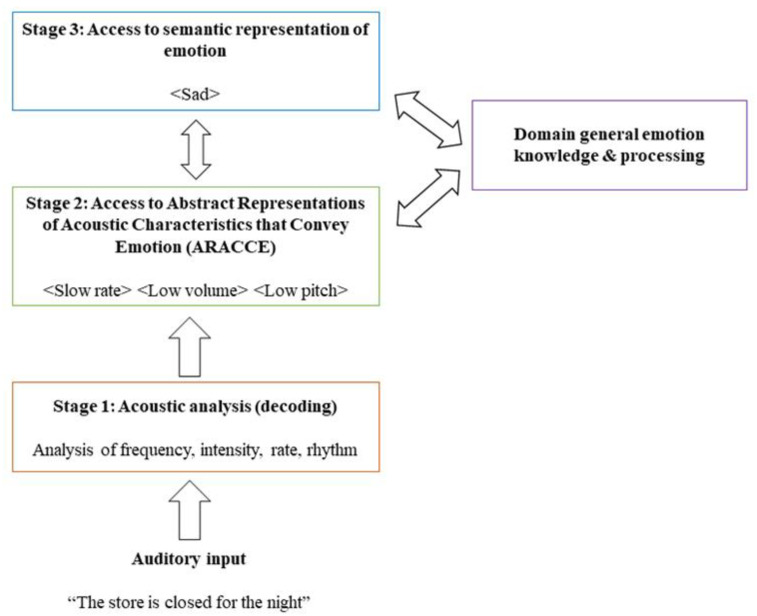
Model of affective prosody recognition as described by Wright et al. [2] and further refined by Sheppard et al. [23]. The model includes the three proposed stages underlying affective prosody recognition as well as an interactive loop between Stages 2 and 3. A description and example processing is included at each model stage. Not yet considered in the model are domain-general cognitive processes (e.g., attention, executive functioning, working memory) that interact and support successful affective prosody recognition.

Thus, it appears that aprosodia and aphasia share gross network organizational features across the right and left hemispheres, respectively. It would follow that even finer specialization may occur for distinct affective prosody recognition subprocesses such as that observed in propositional language (e.g., lexical selection, phonological decoding, etc.). Leading models of affective prosody recognition broadly divide subprocesses into sensory/perceptual extraction (Stage 1), sensory/perceptual-conceptual integration (Stage 2), and cognitive evaluation (Stage 3) [2,13,14,23,25]. The model proposed by Wright and colleagues [2], and further refined by Sheppard and colleagues [23] describes a three-stage model of receptive affective prosody that elaborates upon how acoustic-prosodic features are mapped onto semantic representations of emotions. Stage 1 comprises perceptual processing in the form of acoustic-prosodic decoding from the incoming auditory message. Stage 2 includes perceptual-conceptual integration wherein profiles of acoustic-prosodic features are matched to listeners’ expectations of how given emotions “sound”. These expectations of prosodic variation by emotional expression are termed abstract representations of acoustic characteristics that convey emotion (ARACCE). ARACCE are comparable to the lemma stage of lexical retrieval in single word auditory comprehension models [26,27] or lexical orthographic representations in reading and spelling [2] and mediate the relationship between bottom-up acoustic-prosodic information with top-down conceptual emotion information. The final stage of the model (Stage 3) comprises access to a semantic representation of the expressed emotion. General emotion processing flows between Stages 2 (ARACCE) and 3 (conceptual emotion representations). Figure 1 presents a diagram of this receptive affective prosody model.

One case series [2] demonstrated that adults with aprosodia can have selective damage to various underlying subprocesses. Three adults in the study had receptive aprosodia. Two of these three adults had difficulty identifying prosodic features (e.g., pitch, rate) from sentences spoken with varying affective prosody but performed relatively well when asked to describe the prosody of different emotions. These two cases had difficulty with perceptual/sensory processing (Stage1) but did not have difficulty integrating sensory and conceptual information (ARACCE access; Stage 2). A third case of receptive aprosodia in an individual with early frontotemporal dementia had relatively intact perceptual/sensory processing but impaired ARACCE access for recognition as demonstrated by the difficulty matching acoustic-prosodic features to various emotions. Since behavioral impairments corresponding to different loci can contribute to observed receptive aprosodia, it is posited that distinct regions within the right hemisphere ventral stream are recruited for subprocess computation, and that focal damage disrupts computation within or between subprocesses. In the same case study series by Wright and colleagues [2], the two individuals with impaired acoustic-prosodic extraction (Stage 1 deficits) had lesions encompassing the right anterosuperior temporal lobe, insula, and amygdala. The individual with frontotemporal dementia who demonstrated impaired ARACCE access for recognition had diffuse right hemisphere atrophy, so a focal site of damage underlying that impairment could not be identified.

There is emerging evidence to support distinct lesion loci resulting in selective deficits of underlying affective prosody recognition processes. Sheppard and colleagues [23] investigated the lesion-symptom relationship of affective prosody recognition and its related (sub)processes in a group of 20 adults with acute receptive aprosodia due to RHD. Cluster analyses of behavioral performance revealed three patient groupings. One group had relatively mild receptive aprosodia with impaired ARACCE access (Stage 2) and intact recognition of emotional facial expressions with lesions predominantly in frontotemporal regions. Another group appeared to have general emotion recognition deficits as evidenced by the most severely impaired recognition of emotion in prosody and facial expressions with damage sustained primarily to basal ganglia and subcortical white matter. The third group demonstrated impaired acoustic feature extraction in tones, and visual inspection of lesion location appeared to maximally overlap in occipitotemporal as well as subcortical (thalamus, basal ganglia) regions. Modeling performance on the acoustic feature extraction in tones task resulted in significant predictors of damage to the right middle frontal gyrus and degree of hypoperfusion to the parietal territory of the right middle cerebral artery. Taken together, it appears that subcortical right hemisphere lesions may result in severe, multimodal emotion recognition deficits and that impaired acoustic extraction (Stage 1) was associated with more significant receptive aprosodia compared to impaired ARACCE access (Stage 2).

There are two main benefits to investigating communication lesion-symptom relationships in acute stroke. First, investigators can assess performance before substantial neural reorganization occurs, facilitating the interpretation of impaired tissue and subsequently impaired behavioral performance. Second, the impact of not only infarct but also decreased blood flow on affective prosody recognition can be better appreciated when accounting for hypoperfusion as we did in the current study. Subcortical lesions, such as those in the basal ganglia, can coincide with the cortical hypoperfusion and subsequent behavioral deficits (e.g., [28]). Once cortical tissue is reperfused, improvement in language function has been observed [28,29]. Thus, impaired behavioral performance may not be a result of infarct but also the interplay of a subcortical infarct and subsequent reduced cortical blood flow [29,30,31]. It is important, whenever possible, to consider both infarct and hypoperfusion when investigating acute lesion-symptom relationships as understanding of each one’s contribution can have significant implications when tracking the trajectory of recovery over time. Perfusion-weighted magnetic resonance imaging and computed tomography perfusion are superior for mapping dysfunctional tissue caused by hypoperfusion, but not all patients receive these imaging methods as part of their routine clinical care. Estimates of hypoperfusion can be completed as discussed later in our study. Of course, these estimates are not as sensitive as hypoperfusion data obtained from perfusion imaging, but it is better than the alternative of not accounting for hypoperfusion.

As more information on the lesion and behavioral components that impact post-stroke affective prosody recognition is learned, more theoretically motivated and empirically based behavioral interventions can be developed. There has been promising work into expressive aprosodia treatment for right hemisphere stroke and acquired brain injury. Treatment developed by Rosenbek [32,33] and Leon [34] and colleagues uses two different approaches to target affective prosody production. The motoric approach focuses on repetition of affective prosody with varying contextual support while the cognitive-linguistic approach integrates conceptual components (i.e., corresponding facial expression, description of affective prosody, and the target emotion of the trained prosody) to improve production. The cognitive-linguistic approach may also implicitly improve affective prosody recognition since it taps into higher-level affective and prosodic processes. Recognition performance after expressive aprosodia training was inconsistent across participants and tasks [34].

There is a dearth of evidence supporting the behavioral interventions for receptive aprosodia following RHD. McDonald and colleagues [35] investigated an emotional prosody treatment program following acquired brain injury, but only 20% of the participants (4/20) had damage isolated to the right hemisphere only, and only one individual who was on the waiting list (control) group experienced RHD due to stroke. Interventions for affective prosody recognition, or, more generally, social cognition or emotion perception that included prosody [36], have been investigated in schizophrenia/schizoaffective disorder [37], acquired brain injury [35,38,39,40,41], and autism spectrum disorders [42]. Some interventions addressed receptive aprosodia as part of a larger training program or indirectly through social cognition training [38,39,40,41,42,43]. Methodology (e.g., online vs. one-on-one vs. group or affective prosody targeted directly vs. indirectly) and dosage vary widely across these studies and likely contribute to the mixed outcomes observed. Outcome measures employed by these treatment studies frequently assess affective prosody recognition more contextually, which does not help to elucidate the mechanisms or underlying processes of affective prosody recognition that contribute to the observed deficits nor what aspect(s) of the intervention was/were successful to improve the recognition abilities.

Lado-Codesido and colleagues [37] directly targeted receptive aprosodia in individuals with schizophrenia or schizoaffective disorder using a computer program called *Voices.* The program included eight sessions (two sessions a week), and each session was about 30 min in length. Sessions comprised participants listening to simple, emotionally neutral phrases (as many times as desired) and choosing the emotion conveyed by the recorded speaker. As the program progressed, sessions became more challenging by increases in the number of sentence stimuli to be evaluated, the number of emotion response options provided, and the emotion complexity. The group that completed standard treatment + *Voices* training had significantly better prosody recognition scores on a separate prosody measure compared to the individuals who received standard treatment alone. McDonald and colleagues [35] also targeted affective prosody recognition in a group of 20 individuals with acquired brain injury. Their training protocol comprised three two-hour sessions using dyadic game structures paired with effective brain injury rehabilitation techniques (e.g., errorless learning) in increasingly complex emotional scenarios. Though no treatment effects were observed at the group level, the researchers observed that six of the ten individuals in the treatment group improved on the primary outcome measure while none of the participants in the control group showed improvement. Additionally, five of the six individuals who improved on the primary outcome measure demonstrated evidence of clinically significant improvement; however, this finding was not statistically significant from the control group.

Though hemispatial visual neglect is commonly cited as a hallmark symptom of acute RHD, receptive aprosodia is more common acutely [44] but remains under-diagnosed [45]. Additionally, about a third of adults identified with receptive aprosodia acutely continue to have difficulties chronically [15]. Management of receptive and expressive aprosodia falls uniquely within the scope of practice of speech-language pathology, but many speech-language pathologists (SLPs) feel ill-equipped to assess and/or treat such cognitive-communicative difficulties, likely resulting in many adults with unaddressed receptive aprosodia impairments [46]. Early targeted intervention after stroke may help reduce the number of individuals struggling with receptive aprosodia later in their recovery when reintegration into social, familial, and professional roles is common.

### Research Questions and Hypotheses

The current study investigated the effectiveness of a brief novel explicit acoustic-prosodic-emotion training on affective prosody recognition in acute RHD due to stroke with the aim of answering four research questions:

1. Does affective prosody recognition improve following acoustic-prosodic-emotion recognition training?
  Since training tasks target Stages 1 and 2 of affective prosody recognition, it is expected that recognition will improve after training in individuals with deficits at these processing stages.
2. Does behavioral impairment locus impact training effectiveness?
  If Stage 1 (perceptual) deficits are related to more severe prosody recognition impairments, then these individuals are predicted to benefit more from training compared to individuals with Stage 2 (conceptual) deficits that result in less severe affective prosody recognition impairments.
3. Are distinct lesion loci observed for different behavioral impairment loci?
  Based on previous work and models of receptive aprosodia, distinct lesion loci are predicted for Stage 1 and 2 deficits. Maximal lesion overlap is predicted to occur within posterior and subcortical ventral stream regions (e.g., superior posterior temporal gyrus, basal ganglia, thalamus) for Stage 1 deficits and within more frontal ventral stream regions (e.g., inferior frontal lobe) for Stage 2 deficits.
4. Does degree of impaired tissue (acute lesion and/or hypoperfusion) predict affective prosody recognition training effectiveness?
  It is hypothesized that more severe damage and hypoperfusion to right hemisphere ventral stream/subcortical regions will result in worse deficits in prosody recognition, and thus, greater benefit from training.

## 2. Method

### 2.1. Participants

Informed consent was obtained for all subjects involved in the study. The study was conducted according to the guidelines of the Declaration of Helsinki and approved by the Institutional Review Board of Johns Hopkins University (NA_00042097, continuing review approved 14 January 2021). The records of 185 adults with RHD due to acute ischemic stroke were screened for the current study. These participants were recruited as part of a larger longitudinal study. Only individuals who completed all affective prosody recognition and training tasks were screened for inclusion/exclusion criteria. Assessments and training occurred over the course of a single session or multiple sessions depending on patient fatigue and availability between routine hospital care.

Inclusion/exclusion criteria were evaluated via medical record review and personal interview. Adults with RHD were included if they were premorbidly proficient in English. Individuals were excluded if they had uncorrected vision (except for hemispatial visual neglect) or known hearing impairment; a history of psychological disorders or neurological disease/injury except for prior asymptomatic stroke, lacunar infarct, or transient ischemic attack; or if their acute stroke extended into or involved the left hemisphere. Hemispatial visual neglect was assessed using a combination of tasks, including letter and shape cancellation, scene copy, and line bisection. If evidence of neglect was observed, subsequent testing materials were presented within participants’ right visual field; otherwise, testing materials were placed at midline. Acute behavioral testing was conducted in participants’ hospital room.

Thirty-eight adults with acute RHD (14 female, 24 male; 16 African American, 22 Caucasian; 31 right-handed, 5 left-handed, 2 unknown) were identified for the study. Participants were 63.7 ± 13.3 years of age (28–87) and had 14.0 ± 3.6 years of education (7–22). Behavioral testing was initiated within 3.3 ± 1.9 days (1–7) and a routine clinical MRI of the brain was obtained for 35/38 participants within 1.2 ± 1.5 days (0–7) of hospital admission for acute stroke. Average National Institute of Health Stroke Scale score (NIHSSs) upon hospital admission, an indication of overall stroke severity, was 6.1 ± 5.7 (0–18).

#### Diagnosing Receptive Aprosodia and Determining Locus of Impairment

To determine the presence of receptive aprosodia before training as well as locus of impairment among participants with RHD, their performance on the affective prosody recognition and the three training tasks was compared to the performance on the same tasks completed by healthy, typically aging adults without acquired brain damage (*n* = 29; 14 female, 15 male; 23 Caucasian, 2 African American, 4 unknown; Age: 60.8 ± 14.5 years (28–83); Education: 16.8 ± 3.0 years (12–22); 27 right-handed, 2 left-handed). The clinical and control groups did not differ in age (*t*(65) = −0.840, *p* = 0.404) or sex (*X*^2^(1) = 0.884, *p* = 0.347), but controls demonstrated higher educational attainment compared to the participants with RHD (*t*(62) = 3.387, *p* = 0.001). Presence of receptive aprosodia and locus of impairment were determined if participants’ scores on pre-training affective prosody recognition and training tasks, respectively, fell at or below the fifth percentile (*z* = −1.645) of scores from healthy adults. For acoustic feature training, control participants were all at ceiling, so scores below 100% were considered impaired. An acoustic-prosodic impairment locus was determined based on the performance on acoustic and prosody feature training tasks. An ARACCE impairment locus was determined based on the performance on the ARACCE training task. Participants could demonstrate impairment loci at either Stage 1, Stage 2, or Stages 1 and 2.

### 2.2. Stimuli and Procedures

#### 2.2.1. Affective Prosody Recognition

To collect baseline data and to determine the presence of receptive aprosodia, participants first judged the affective prosody conveyed by sentences containing either real words (with emotionally neutral content, see Table S1 for a list of sentences) or pseudowords [47,48]. Real-word and pseudoword sentences were recorded by two female speakers, one speaker for each sentence type. Slightly more than half of participants (20/38) were presented with pseudoword sentences while the remaining 18 were presented with real-word sentences. The pseudoword sentences contained English pronouns (e.g., I, they), articles (i.e., a, an, the), and prepositions (e.g., on, under) but included noun and verb non-words (e.g., wanced, nonitor). Sentences were presented auditorily only once unless the test administrator judged that external distractions (e.g., physician or nurse entering patient’s room for care) warranted replaying of the sentence. Written emotion options (i.e., angry, sad, happy, surprised, afraid, bored) were arranged vertically in the middle of a laptop computer screen and remained visible throughout the task. Task instructions were presented visually and auditorily to participants. Affective prosody recognition was assessed before and after the completion of the three training tasks (described next). No feedback was provided, and sentences were presented at a comfortable volume as determined by the participant.

Five emotions (i.e., happy, sad, angry, afraid, surprised) were presented in both real-word and pseudoword sentences. Recognition of surprised prosody was assessed in both linguistic contexts, but the type of surprise differed between them (pleasant vs. shocked), and training focused on shocked rather than pleasant surprise. Additionally, boredom was presented in real-word sentences only. Therefore, planned analyses were conducted on the recognition of happy, sad, angry, and afraid emotions.

#### 2.2.2. Acoustic Feature (Stage 1) Training

Participants were presented with pairs of pure tone sequences. Sequence pairs varied by only one acoustic feature (i.e., rate, duration, volume, pitch). Acoustic features were presented orthographically in a multiple-choice format and arranged horizontally in the center of a laptop computer screen. Acoustic features were also read aloud to the participants as needed per the test administrator judgment. Participants were asked to select which acoustic feature differed between the sequences. Visual and verbal feedback was provided for correct and incorrect responses. After all tone sequence pairs were presented, pairs that were judged incorrectly by participants were repeated, with feedback again provided for correct and incorrect responses. This procedure was repeated once more for any remaining incorrect responses for a possible total of three presentations of each tone sequence pair. Tone sequence pairs were played only once per presentation unless external distractions were judged to interfere with the presentation per test administrator judgment. Scores from the final presentation were recorded and used in the determination of impairment locus. For example, if a participant scored 3/9, 5/9, and 8/9 for each presentation of tone sequence pairs, the score recorded would be 8/9. Likewise, if a participant scored 7/9 and then 9/9, then there would not be a third presentation, and the score recorded would be 9/9. Tone sequence pairs were presented at a comfortable volume as determined by the participant.

#### 2.2.3. Prosodic Feature (Stage 1) Training

After completing the acoustic feature training, participants completed the prosodic feature training with the same list of acoustic features (i.e., rate, duration, volume, pitch). Sentences comprised real words conveyed with varying affective prosody (i.e., happy, sad, angry, afraid, shocked surprised, bored). Instead of asking participants to identify the emotion conveyed in the speaker’s tone of voice, participants were asked to identify two or three prosodic features of the speech in a forced-choice format (i.e., Fast rate vs. Slow rate? High pitch vs. Low pitch? Loud vs. Quiet? Flat pitch vs. Rising pitch?). Prosodic features were presented orthographically and vertically oriented at the center of a laptop computer screen. Prosodic features were also read aloud to participants as needed per the test administrator judgment. Visual and verbal feedback was provided for correct and incorrect responses. Sentences were played only once unless external distractions were judged to interfere with the presentation as per the test administrator judgment. Sentences were presented at a comfortable volume as determined by the participant.

#### 2.2.4. ARACCE (Stage 2) Training

To target ARACCE access and processing, participants were presented with an emotion (i.e., happy, sad, angry, afraid, shocked surprised, bored) orthographically at the top of a laptop computer screen and asked to identify two or three prosodic features that described the tone of voice associated with the emotion (e.g., Sad = <slow rate> <quiet> <low pitch>) [49]. Prosodic features were presented orthographically, arranged vertically in the center of a laptop computer screen below the emotion, and comprised the features trained during the acoustic-prosodic training tasks. Features were read aloud to participants as needed per the test administrator judgment. Visual and verbal feedback were provided for correct and incorrect responses.

Feedback provided during training included both knowledge of results (correct vs. incorrect) and knowledge of performance (providing the correct/target response to participants). Knowledge of performance appears to demonstrate greater motor recovery compared to the knowledge of results (e.g., [50]), but both are found to be effective (e.g., see [51] for a discussion). The effect of feedback type on conceptual learning is not yet clear. Affective prosody recognition and training tasks were completed in a single session or across multiple sessions as required due to participant’s fatigue and schedule.

### 2.3. Imaging

Acute MRI scan sequences were acquired on 1.5 and 3.0T Siemens scanners as part of the routine clinical care. Diffusion-weighted imaging (DWI) sequences were used to visualize and manually trace acute lesions. Fluid attenuated inversion recovery (FLAIR) sequences were referenced to estimate the volume of hypoperfusion via ratings of presence and severity of hyperintense vessels, which has been found to significantly correlate with the volume of hypoperfusion on perfusion-weighted imaging [52]. T2-weighted imaging was completed to check for additional structural abnormalities or lesions. Susceptibility-weighted imaging (SWI) was referenced to rule out the presence of acute hemorrhage. Lesion tracings were completed manually using MRIcron [53,54] by a trained technician (AZD) and supervised by a neurologist (AEH). These tracings were then normalized to MNI space via SPM12 [55]. Normalization was completed on DWI images using a template based on healthy older adults [56], and the warping parameters were then applied to the lesion traces. The proportion of acute lesion volume per region of interest (ROI) was calculated separately for each participant using the automated anatomical label (AAL) [57] for gray matter regions as well as the tractography-derived white matter tract atlas (CAT) [58] for white matter regions.

Since perfusion-weighted [MR] imaging (PWI) or CT perfusion was not available for all participants, FLAIR hyperintense vessel ratings for the right cerebral hemisphere were completed by a trained technician (LB) blinded to behavioral prosody performance and supervised by a neurologist (AEH). Ratings on the scale range from 0 (no hyperintense vessels present) to 2 (3+ hyperintense vessels present on 1 slice, or 3+ slices with presence of hyperintense vessels) and are given to six different regions of the brain determined by blood supply (posterior cerebral artery (PCA); anterior cerebral artery (ACA); middle cerebral artery frontal (MCA-Frontal), parietal (MCA-Parietal), insular (MCA-Insular), and temporal (MCA-temporal) regions). Each point equates to about 16 mL (or 16,000 mm^3^) of hypoperfused tissue [52].

### 2.4. Analyses

R (v3.6.3) was used to complete all statistical analyses. Linear mixed effects modeling was employed to investigate the effect of training and behavioral impairment locus on affective prosody recognition performance among participants with receptive aprosodia subsequent to acute RHD (Q1 and Q2). Time point (pre-training vs. post-training), impairment locus (acoustic-prosodic vs. ARACCE vs. ARACCE + acoustic-prosodic vs. none), and linguistic context (pseudoword vs. real-word sentences) were included as predictors. During preliminary inspection of data plots, we observed that accuracy on real- and pseudoword sentences differed, so linguistic context was included as a predictor in the linear mixed effects model. Affective prosody recognition accuracy (percentage) on happy, sad, angry, and afraid sentences was assessed pre- and post-training and served as the outcome variable. The effects of age, education, and NIHSSs were assessed as possible covariates during model selection, and participants were modeled as random intercepts. Model selection was determined by comparing Akaike Information Criterion (AIC) values as well as by Likelihood Ratio Tests. lme4 [59] and lmerTest [60] packages were used for linear mixed effects modeling.

Lesion subtractions maps were created to investigate the lesion profiles for each behavioral impairment locus (Q3) using MRIcron [53,54]. Finally, Least Absolute Shrinkage and Selection Operator (LASSO) regression was employed to investigate the contribution of damage sustained to specific right hemisphere ROIs due to infarct and/or hypoperfusion on affective prosody training effectiveness (Q4). LASSO regression methods are ideal for analyses comprising small sample sizes, many possible predictors, and multicollinear predictors that allow for concurrent variable selection and regression [61]. LASSO serves as both a variable selection and regression model tool. This method utilizes a shrinkage parameter to result in a single, sparse, optimally predictive model. Percent damage (calculated for the right-hemisphere only using NiiStat (https://www.nitrc.org/projects/niistat/) (accessed on 19 May 2021) via Matlab (MathWorks, vR2020a) using AAL [57] and CAT [58] atlases) and hyperintense vessel ratings were entered into the model if at least two participants had damage to each region, resulting in the inclusion of 17 ROIs previously associated with receptive prosody [2,9,15,16,17,18,19,20,21,62]: inferior frontal gyrus pars opercularis, insula, supramarginal gyrus, angular gyrus, caudate, putamen, globus pallidus, thalamus, Heschl’s gyrus, superior/middle/inferior temporal gyri, superior temporal pole, and internal capsule. White matter tracts within the ventral stream (inferior longitudinal fasciculus (ILF), inferior fronto-occipital fasciculus (IFOF), and uncinate) that have not been specifically implicated in affective prosody recognition were also included. Age, education, total lesion volume, pre-training affective prosody recognition score, and degree of hypoperfusion to rACA and each rMCA (frontal, insular, parietal, temporal) distribution were also entered into the model using leave-one-out cross validation LASSO (5000 permutations).

## 3. Results

### 3.1. Participants with RHD and Receptive Aprosodia

Roughly 47% (18/38) of the participants with acute RHD presented with affective prosody recognition impairments. Compared to the individuals with RHD who were not aprosodic, individuals with receptive aprosodia were older (*t*(31.439) = −2.731, *p* = 0.010). Education (*t*(33) = 0.727, *p* = 0.472) and sex (*X*^2^(1) = 0.181, *p* = 0.671) did not differ between the two groups. Of the participants with receptive aprosodia, almost 40% (7/18) demonstrated impaired acoustic-prosodic recognition (Stage 1), about 5% (1/18) demonstrated impaired ARACCE access (Stage 2), 33% (6/18) were impaired at both Stages 1 and 2, and about 22% (4/18) did not demonstrate acoustic-prosodic- or ARACCE-specific processing deficits. Additional information about participants with receptive aprosodia can be found in Table 1. Since only one individual was identified as demonstrated impaired ARACCE access (Stage 2), individuals with Stage 2 and individuals with Stages 1 and 2 deficits were combined into one group for subsequent statistical analyses.

### 3.2. Affective Prosody Recognition Training Effectiveness (Q1, Q2)

Participants identified with receptive aprosodia at acute testing as detailed in *Diagnosing Receptive Aprosodia and Determining Locus of Impairment* were included in the linear mixed effects model (18 participants, 36 data points). Affective prosody recognition percent accuracy served as the predicted (dependent) variable. First, the random effect (participants) and covariates (i.e., age, education, NIHSSs) were added to create the first covariate model. Inclusion of education and NIHSSs along with the random effect resulted in the lowest AIC value and thus was selected as the final covariate model. Next, all fixed effects (locus of impairment, time point (i.e., pre-training, post-training), linguistic context) were added as main effects. This model was then compared to a model with the additional interaction term impairment locus × time point in order to investigate if behavioral impairment locus differentially impacted training effectiveness. There was no statistically significant difference between the models (*p* = 0.945), so the main effect-only model was selected since its AIC value was lower and it was a simpler model. Linear regression and generalized estimating equation models containing the same fixed effects as the full linear mixed effects model were also built to assess the best model fit. The linear mixed effects model (with only main effects) was found to have the lowest AIC value and was selected for the final model interpretation. AIC-corrected values were also checked since the ratio of participants to predictors was small (e.g., see [63]); AIC-corrected values were found to mirror uncorrected values, so uncorrected AIC values were reported. Linear mixed effects model assumptions were checked and were met.

**Figure 2:**
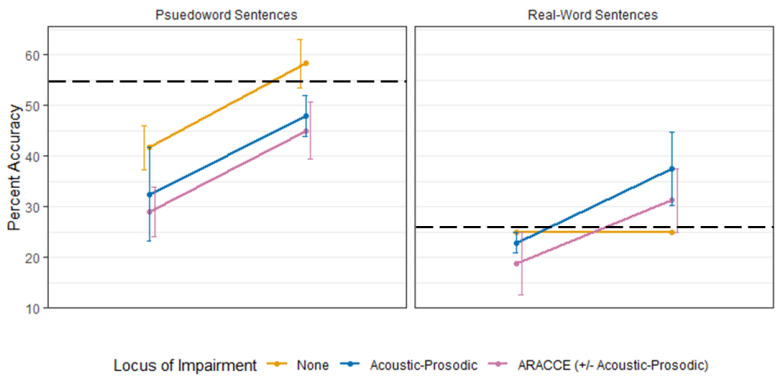
Mean and standard error of affective prosody recognition performance before and after training by behavioral impairment locus. Error bars are dodging so that full range can be appreciated for each group without interference of overlapping error bars. There were significant main effects of linguistic context (real-word < pseudoword sentences) and testing (pre-training < post-training) but no significant effect of behavioral impairment locus. The horizontal dashed black line represents the cutoff accuracy for impairment based on controls’ affective prosody recognition performance at pre-training.

The final model was statistically significant compared to the covariate-only model (*p* < 0.001, marginal *R*^2^ = 0.521). There was a significant main effect of time point, with a roughly 14% increase in accuracy after training compared to before training (*p* < 0.001). There was also a main effect of task. Affective prosody recognition accuracy in real-word sentences was about 12% lower compared to the accuracy in pseudoword sentences (*p* = 0.003). There was no significant effect of behavioral impairment locus (*p* > 0.250). See Table 2 for a summary of the model selection process, Table 3 for a summary of the final model, and Figure 2 for mean affective prosody recognition performance by impairment loci before and after training.

### 3.3. Lesion Contributions to Affective Prosody Recognition Training (Q3 and Q4)

Seventeen of the 18 participants with receptive aprosodia and acute RHD had an MRI of the brain. Formal statistical comparisons of infarcted right hemisphere regions by behavioral impairment loci were deemed inappropriate due to small group sizes. Instead, lesion clusters were explored qualitatively using subtraction maps, wherein lesions unique to a behavioral impairment locus are displayed by creating lesion overlap maps for that group and removing lesions associated with the other groups.

**Figure 3:**
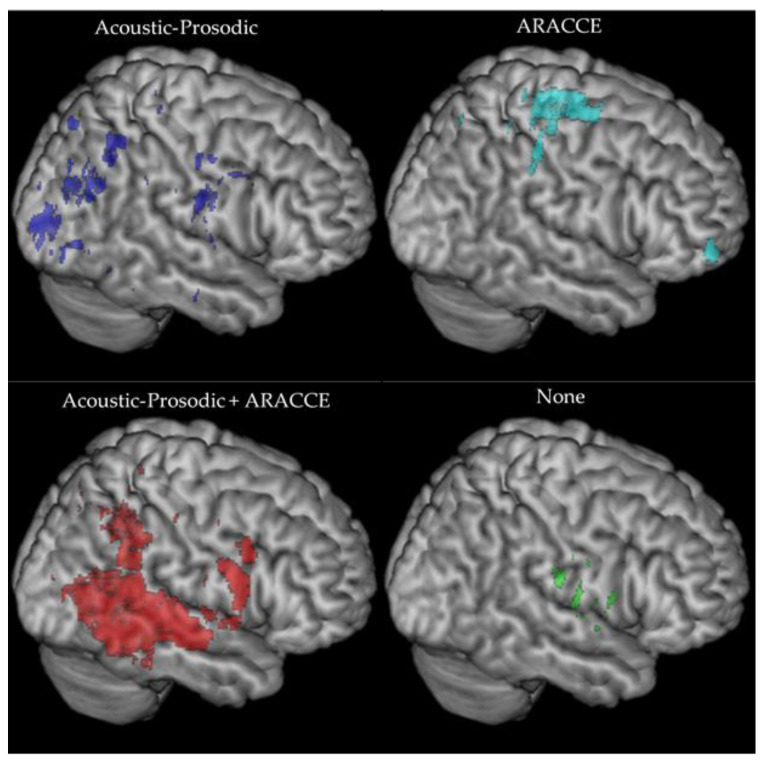
Lesion clusters for participants with receptive aprosodia characterized by acoustic-prosodic (**top left**), ARACCE (**top right**), acoustic-prosodic + ARACCE (**bottom left**), and no impairment loci (**bottom right**).

All groups demonstrated some subcortical involvement (i.e., basal ganglia, thalamus, deep white matter tracts), albeit to different degrees. Participants with receptive aprosodia but who did not clearly demonstrate a behavioral impairment locus (*n* = 4) had a small cluster of inferior frontal lesions. Participants whose receptive aprosodia was characterized by acoustic-prosodic deficits (Stage 1; *n* = 7) had primarily occipitoparietal and subcortical (basal ganglia, thalamus, internal capsule) lesions. Acoustic-prosodic + ARACCE deficits (Stages 1 and 2; *n* = 5) were associated with temporoparietal and frontotemporal regions, comprising the angular and supramarginal gyri, arcuate fasciculus, inferior frontal gyrus pars opercularis, and temporal lobe. Finally, one participant whose receptive aprosodia was characterized by impaired ARACCE access (Stage 2) alone demonstrated a unique lesion profile comprising the middle orbitofrontal gyrus, supplementary motor area, and paracentral lobule. Figure 3 presents the lesion clusters for each behavioral impairment group.

LASSO regression analyses on affective prosody recognition accuracy differences (post-training–pre-training) yielded a significant model (lambda = 0.858, *p* < 0.05) comprising eight predictors: age, education, degree of hypoperfusion to ACA and MCA-parietal regions as well as percent damage sustained to the angular gyrus, thalamus, ILF, and IFOF. Older age, less education, and greater degree of lesioned tissue was associated with less gain in affective prosody recognition scores following training. No predictor was found to be independently significant. Output of the LASSO regression can be found in Table 4.

**Figure 4:**
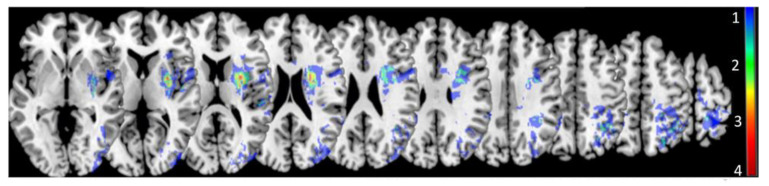
Lesion subtraction map of participants with RHD who were still considered aprosodic after acoustic-prosodic-emotion training. Color gradient scale on the right side of the image indicates the number of participants with overlapping lesions.

A lesion subtraction map was created to help visualize right hemisphere regions with greatest lesion overlap in individuals with RHD still considered aprosodic after training (*n* = 11) and included basal ganglia and subcortical frontal and parietal white matter regions (Figure 4).

## 4. Discussion

The current study aimed to investigate the effectiveness of a brief novel explicit training for receptive aprosodia following acute RHD. Training targeted both perceptual (acoustic-prosodic) and conceptual (ARACCE) processes proposed to underlie accurate, automatic prosodic decoding. Training was conducted in adults with acute RHD due to stroke, which commonly precipitates affective prosody recognition deficits [15,44], more so than at later stages of recovery.

### 4.1. Behavioral Contributions to Affective Prosody Recognition Training (Q1 and Q2)

Our initial question was, “Does affective prosody recognition improve following acoustic-prosodic-emotion training?” Affective prosody recognition significantly improved after explicit training in participants with receptive aprosodia, supporting the first hypothesis. Training focused on perceptual processes (i.e., recognition of acoustic features (rate, pitch, volume, duration) in pure tone sequences and sentences spoken with varying affective prosody) and conceptual processes (i.e., matching prosodic features to emotion “profiles”) proposed to underlie successful affective prosody recognition. According to the model outlined by Wright and colleagues [2] and expanded upon by Sheppard and colleagues [23], the conceptual stage, termed ARACCE access (Stage 2), can be described as an emotion lexicon that shares similarities with linguistic models including lemma stages [26,27] and lexical orthographic representations [2].

Second, we asked if behavioral impairment locus would impact training effectiveness. There were no observed group effects of behavioral impairment locus on training, resulting in the rejection of the second hypothesis. Participants with Stage 1 (perceptual; acoustic-prosodic recognition), Stage 2 (conceptual; ARACCE access), Stages 1 and 2, and no observed behavioral impairment locus appeared to benefit similarly from training. Previous work has suggested that Stage 1 (perceptual) deficits were associated with more severe receptive aprosodia [23] and thus would potentially benefit more from training. However, this pattern was not observed in the current study. It is not completely surprising that no difference was observed between groups since training targeted both acoustic-prosodic and ARACCE access. Though a single session of training improved affective prosody recognition on a group level, individual-level performance varied widely. Over 75% of participants with receptive aprosodia (13/18) increased their recognition accuracy after just the one session of training, but about 60% (11/18) were still considered aprosodic after training. Recognition improvement following one training session may serve to identify “good” responders (i.e., stimulability) to further training sessions targeting acoustic-prosodic-emotion recognition. That is not to say that individuals whose affective prosody recognition did not improve with one session would not also benefit from additional training. Perhaps modifications to the dosage/frequency would be necessary for less stimulable participants to show improvement. The possibility of predicting good candidates for this type of treatment and determining the dose/frequency to achieve long-term retention requires further investigation. Likewise, it is possible that these individuals (who did not improve with training) would benefit from a completely different, perhaps implicit, or more contextual, approach.

Generally, participants’ affective prosody recognition accuracy improved across groups following acoustic-prosodic training. However, not all participants improved to performance ranges of healthy adults (above the black dashed line in Figure 2), and this finding differed by linguistic context. For pseudo-word sentences, only participants without a measured behavioral impairment locus improved to functional range while participants with acoustic-prosodic and participants with ARACCE ± acoustic-prosodic deficits improved but were still considered impaired. Interestingly, the opposite pattern was observed in real-word sentence recognition post-training: participants with acoustic-prosodic deficits and participants with ARACCE ± acoustic-prosodic deficits improved to control-level performance while participants without a measurable impairment locus did not improve. Our hypothesis as to why participants without acoustic-prosodic impairment loci did not improve to within functional limits (above the cutoff for aprosodic) is that the single training episode was not effective to improve recognition abilities based on their underlying difficulty with affective prosody recognition. For instance, it may be the case that these individuals have difficulty integrating Stage 1 and 2 deficits that, when trained in isolation as in the case of the training tasks, they do not appear impaired on the individual task. Rather, they have a difficulty integrating that information together into a coherent process that supports accurate affective prosody recognition. This difficulty integrating processes or information from multiple steps may indicate a cognitive impairment component, but this supposition is only speculative in the current study since we did not assess cognitive abilities (e.g., attention, recall, working memory, executive functioning, etc.,) and their relationship to receptive aprosodia as part of this study. Implementation and integration of training tasks into affective prosody recognition did not appear to carry over for these individuals.

Clearly, a single session of explicit training underlying perceptual and conceptual processes is not effective to improve vocal emotional recognition for all individuals with receptive aprosodia following acute RHD. Age does not appear to significantly impact the affective prosody recognition gains from pre- to post-training (*r_p_* = 0.078, *p* = 0.766). The linear mixed effects model included a significant independent predictor of context, with affective prosody recognition accuracy higher in pseudoword sentences compared to real words. It is posited that the pseudoword sentences required a lower demand on cognitive resources since they carried relevant prosodic information only. In comparison, real-word, semantically neutral sentences carried both relevant prosodic and linguistic information, albeit not lexical-semantic information that likely bootstrapped receptive prosody. Therefore, all cognitive faculties (e.g., attention, memory, auditory-verbal processing) might be better focused on prosody rather than linguistic decoding/comprehension in pseudoword sentences. Conversely, real-word sentences may introduce the possibility of dividing neural resources across cognitive domains, thus taxing the cognitive-communicative system beyond its capabilities. Under the cognitive resources hypothesis, participants with RHD have less cognitive faculties available due to brain damage, so fewer or inefficient allocation of resources contributes to deficient processing [64]. Though less ecologically valid, pseudoword sentences may serve as an ideal context, at least in acute stages, for adults with RHD and receptive aprosodia to focus on prosodic recognition without the influences, whether facilitative or interfering, of linguistic information. Since linguistic context was included in the model due to preliminary observation of data, future studies that prospectively manipulate sentential context are needed to better understand the role of context on affective prosody recognition performance.

Despite accounting for linguistic context on affective prosody recognition, the model explained only 50% of the variance in performance. Thus, other factors influencing recognition and training effectiveness must still be at play. Training used explicit, metacognitive methods to teach recognition of important acoustic-prosodic features and matching those features to specific emotions. Metacognitive teaching strategies can be effective therapeutic interventions (e.g., metaphor interpretation) [65,66], but these strategies may tax already-reduced cognitive resources and increase cognitive effort during computation, obscuring true performance in the targeted skill or process [64] as already mentioned. Adults with RHD benefit from contextual supports aimed to decrease the task cognitive demands [67,68,69,70]. For example, Zezinka and Tompkins [71] used a negatively biasing task (e.g., Your breaks don’t work on the freeway. Do you feel afraid or angry?) to increase negative word production during discourse elicitation from a video stimulus [72]. Indeed, after participants completed the biasing task, their production of negative affect words significantly increased. Perhaps by using more implicit cueing or training methods, such as presenting emotional vignettes, emotional scenes, facial expressions, or body language, in conjunction with affective prosody, those implicit supports may scaffold recognition by pairing it with other related emotional processing that may be relatively intact. Since affective prosody recognition deficits are not likely to occur in isolation [23], consideration of how other impaired domains may impact prosodic performance is critical.

Finally, we did not compare the training tasks against each other to determine which training (acoustic-prosodic training (Stage1), ARACCE training, acoustic-prosodic + ARACCE (Stage 1 + 2)) resulted in better affective recognition afterwards. Both sensory and conceptual tasks were included to target two major underlying processes proposed to support affective prosody recognition, which may have impacted our finding of no group differences in recognition. Further development of receptive aprosodia training/treatment approaches would benefit from assessment of what elements or ingredients are most effective in improving performance.

### 4.2. Lesion Contributions to Affective Prosody Recognition Training (Q3 and Q4)

Our third question focused on whether distinct lesion loci would be observed for the different behavioral impairment profiles of the participants. Lesion cluster maps were created to investigate loci differences across behavioral impairment groups (i.e., participants with perceptual (Stage 1), conceptual (Stage 2), perceptual-conceptual (Stages 1 and 2), or no impairment locus). Since group sizes were small, quantitative comparisons could not be appropriately applied; however, qualitative analysis suggests distinct profiles of RHD to corresponding behavioral impairment groups. Only one participant demonstrated impaired conceptual processing (Stage 2) deficits and had damage in the orbitofrontal cortex and superior frontoparietal regions, including the supplementary motor area. A recent meta-analysis [73] observed right supplementary motor area involvement during both linguistic and affective prosody recognition, and this region has also been implicated in socio-emotional perception [74]. Likewise, the orbitofrontal cortex is believed to assist in explicit evaluation of affective prosody [75], which would align more with Stage 2 rather than Stage 1 processing deficits. However, since there was only one individual who met this receptive aprosodia profile, further investigation of individuals with RHD and similar behavioral deficits is warranted to determine a stronger lesion-symptom relationship.

Participants with perceptual deficits (Stage 1) demonstrated more posterior and subcortical lesions compared to the other groups. Their lesions were clustered around occipitoparietal and occipitotemporal regions along with subcortical involvement in the basal ganglia and thalamus. Findings from the current study and Sheppard and colleagues [23] align with previous work demonstrating acoustic-prosodic recognition and posterior (e.g., [15,22]) and subcortical (e.g., [18,20,21,62]) right hemisphere involvement. Participants with perceptual and conceptual deficits (Stages 1 and 2) similarly had two primary lesion loci: one posterior area comprising temporoparietal regions and another more anterior area in the inferior frontal lobe. Both areas have been heavily implicated as hubs for affective prosody recognition [2,14,16,19,22,62,76]. This group of individuals is discussed separately from individuals with Stage 1 or Stage 2 deficits because of their unique lesion profile from the other two impaired groups, suggesting an interesting theoretical distinction in the underlying impaired mechanism. It is posited that individuals with both impairments demonstrate difficulty mapping perceptual information onto conceptual knowledge. That is, there is a breakdown between the translation or transference of acoustic-prosodic decoding to the conceptual emotional lexicon. Posterior middle temporal lesions in the left hemisphere have been associated with impaired lexical and semantic access [11,12], so it follows that this right hemisphere region could play a role in mapping prosodic information to the “emotional lexicon” or ARRACE representations.

The final group of adults with receptive aprosodia had no difficulties with perceptual and conceptual training tasks (i.e., no clear impairment loci). These participants had brain damage within the insula and Heschl’s gyrus. It appears that the right hemisphere regions implicated in the perceptual-conceptual processes underlying affective prosody recognition were relatively spared in this group; however, these participants were still considered aprosodic pre-training. Perhaps cognitive factors, such as reduced cognitive resources (e.g., attention, inhibition, memory), played a role in the observed receptive aprosodia for this group, such as with difficulty integrating processing at each component with the overall task of affective prosody recognition. As mentioned previously, cognitive measures were not acquired on all participants, so this statement is only speculative and requires further systematic investigation to better understand the interplay of cognition and prosody performance in this clinical population. These cognitive processes are currently not included or accounted for in the tested model but should be considered in future model updates and adaptations.

Taken together, hypothesis 3 was partially supported. Right hemisphere brain regions that were previously implicated in affective prosody recognition were also observed in the lesion cluster maps, namely temporoparietal regions, basal ganglia, thalamus, and inferior frontal lobe. However, findings from the current study diverge from previous work in that participants with both perceptual and conceptual deficits present as a unique group separate from individuals with perceptual or conceptual deficits.

The study’s fourth question asked if degree of impaired tissue (infarct and/or hypoperfusion) would predict affective prosody recognition training effectiveness. Findings from the LASSO regression implicated six right hemisphere regions within the ventral stream in affective prosody recognition training gains, supporting the fourth hypothesis (Q4). Percent damage to the angular gyrus, thalamus, white matter tracts (i.e., ILF, IFOF), and rACA and rMCA-parietal hypoperfusion was associated with smaller gains in affective prosody recognition following training. However, no single region emerged as an independent predictor. Of note, pre-training affective prosody recognition accuracy was entered as a possible predictor but was not ultimately selected in the final model.

Inspection of lesion subtraction plots of individuals considered aprosodic after training revealed the greatest overlap in the basal ganglia and adjacent subcortical white matter. Post hoc exploratory group analyses did not reveal any significant differences in demographics, stroke severity, or performance on assessments of semantic representation of emotions or domain-general emotion processing between participants considered aprosodic and those not considered aprosodic after training (all *ps* > 0.050). The basal ganglia play a key role in not only motor-based learning but also cognitive-based learning, including implicit category learning [77,78]. Therefore, individuals with more basal ganglia damage may not capitalize on the categorization of acoustic-prosodic cues and matching those cues to specific emotions as much as individuals without, or with less extensive, damage in this region. It may be worthwhile, then, to investigate the relationship between basal ganglia lesions and the teaching method used during training. As was stated earlier in the discussion, with more severe and more domain-general affect recognition impairment associated with subcortical lesions, perhaps more associative emotional training (e.g., matching the prosody to emotional faces, visual scenes, or short vignettes) would be beneficial to supplement other cueing categorization strategies for individuals with post-stroke receptive aprosodia with basal ganglia involvement.

### 4.3. Limitations and Future Directions

The current study made significant contributions to the literature on acute RHD and receptive aprosodia training, but there are some limitations to consider. First, sample size was relatively small, and future studies of training effectiveness (i.e., stimulability) would benefit from including more participants. Second, the study would have benefitted from collection of more cognitive-linguistic measures to assess the contribution of these variables, or their relationship, to affective prosody recognition. For instance, if it is observed that auditory verbal memory is reduced along with symptoms of receptive aprosodia, then individuals with RHD may benefit from complementary training that targets auditory-verbal memory capacity to boost affective prosody recognition gains. Third and finally, data were limited to acute training during a single training opportunity. Response to this single training session can provide information on participant stimulability for receptive aprosodia treatment, but findings do not address the optimal dosage for effective intervention. Additionally, it is unclear how time after stroke impacts training potency. However, this data provide support for future inquiries into such questions. It will be critical to observe how behavioral impairment profiles change during recovery and if receptive aprosodia training is observed to be more/less effective at different time points and with varying number of training opportunities.

## 5. Conclusions

Brief explicit training focusing on acoustic-prosodic (perceptual) recognition and ARACCE (conceptual emotional lexicon) access is effective to improve affective prosody recognition in adults with acute RHD and concomitant receptive aprosodia. However, this single training session was not as effective for adults with damage to critical right hemisphere ventral stream regions—angular gyrus, thalamus, ILF, and IFOF— and hypoperfusion to ACA and MCA-parietal territories. Additional training or different (implicit) training approaches need to be investigated. The current study provided further support that demographic variables including age and education are important to consider when planning cognitive-linguistic training. Additionally, hypoperfusion appears to contribute to acute receptive aprosodia training effectiveness following stroke. Future work is needed to understand how affective prosody impairments change over time and to determine if the explicit training protocol is effective at later stages of recovery in subacute and chronic receptive aprosodia. Finally, in cases where explicit training is not as effective, perhaps due to the metacognitive task overtaxing available cognitive resources, determining how less demanding, more implicit training can impact recognition performance is needed. Lesion location may provide insight into effective training strategies (implicit vs. explicit) to help individualize and maximize the outcomes for adults with RHD and receptive aprosodia. The preliminary results presented in this study provide an essential foundation for our future research and others, especially because so little work has been published regarding aprosodia (recognition) treatment after right hemisphere stroke.

## Figures and Tables

**Table 1:** Demographic and emotion recognition accuracy (%) of participations with acute RHD and receptive aprosodia.

Participant	Sex	Race	Age	Education (years)	Handedness, Pre-Stroke	Admitting NIHSSs	Affective Prosody Recognition, Pre-Training	Affective Prosody Recognition, Post-Training	Emotion Synonym Matching	Emotional Facial Expression Recognition
STAGE 1 DEFICIST (ACOUSTIC-PROSODIC)										
101	female	white	64	12	right	1	10	58.33	87.50	67.50
102	female	black	85	20	right	1	18.75	37.50	87.50	67.50
105	female	white	75	na	na	15	35	50	70.83	na
107	male	black	56	12	right	4	25	25	79.17	60
108	female	white	76	16	right	8	25	50	91.67	67.50
114	male	white	73	16	left	1	55	41.67	79.17	57.50
117	male	black	70	13	right	6	30	41.67	91.67	52.50
STAGE 2 DEFICITS (ARACCE ACCESS)										
106	male	black	64	18	right	3	45	41.67	91.67	85
STAGE 1 and 2 DEFICITS										
100	female	white	71	10	right	7	20	50	95.83	65
103	female	black	75	12	right	17	20	25	45.83	na
104	male	white	74	10	right	0	25	25	95.83	82.50
110	male	white	63	8	left	3	12.50	37.50	87.50	na
112	male	white	57	na	na	3	35	50	75	82.50
115	male	black	62	10	right	15	25	58.33	54.17	62.50
NO IMPAIRMENT LOCUS										
109	male	white	60	13	right	5	25	25	91.67	65
111	male	black	87	16	right	17	50	66.67	95.83	na
113	male	white	72	16	right	13	40	58.33	95.83	90
116	male	black	63	na	right	10	35	50	70.83	65

NIHSSs = NIH Stroke Scale score; na = not available; ARACCE = Abstract Representation of Acoustic Characteristics that Convey Emotion.

**Table 2:** Summary of base and full model-selection process.

Model Specification	Model Name	Nested Model	Fixed Effects Added	Random Effects	Model Fit				LRT against Nested	
				Subjects	AIC	BIC	LL	df.resid	df	*X* ^2^
Random effect + all covariates	Covariate 1	-	age + education + admitNIHSS	intercepts	298.6	308.1	−143.3	30	-	-
^†^ Random effect + covariate subset 1	Covariate 2	Covariate 1	education + admitNIHSS	intercepts	296.7	304.6	−143.3	31	1	0.129
Random effect + covariate subset 2	Covariate 3	Covariate 2	Education	intercepts	297.8	304.1	−144.9	32	1	3.076
Fixed effects, 2-way interaction	Main effects + Interaction	Main effects	education + admitNIHSS + impairment locus × time + context	intercepts	285.2	302.6	−131.6	25	2	0.113
^†^ Fixed effects, main effects only	Main effects only	Covariate 2	education + admitNIHSS + impairment locus + time + context	intercepts	281.3	295.6	−131.7	27	4	23.343 *

AIC = Akaike Information Criterion; BIC = Bayesian Information Criterion; LL = Log Likelihood; df.resid = residual degrees of freedom; LRT = Likelihood Ratio Tests; df = degrees of freedom; ^†^ = selected models for subsequent statistical comparison; * *p* < 0.00.

**Table 3:** Summary of selected full linear mixed effects model.

Fixed effects					
	**Estimates (Β)**	**SE**	**95% CI**	***t***	***p***
Intercept	22.911	11.215	−17.883, 34.769	2.043	0.048
Impairment locus: Acoustic-prosodic	−5.191	4.546	−15.118, 4.736	−1.142	0.261
Impairment locus: ARACCE ± acoustic-prosodic	−6.168	4.836	−16.727, 4.392	−1.275	0.210
Testing (post-training)	14.468	3.126	7.640, 21.294	4.628	<0.001
Context (real-word sentences)	−12.384	3.821	−20.729, −4.039	−3.241	0.003
Education	1.084	0.610	−0.247, 2.415	1.778	0.084
admitNIHSS	0.063	0.335	−0.668, 0.795	0.189	0.851
Random effects					
	**Variance**			**SD**	
Participant (intercept)	0			0	
Model fit					
	**Marginal**			**Conditional**	
*R* ^2^	na			0.521	

SE = standard error; SD = standard deviation; na = not available; model equation: accuracy (%) ~ Impairment locus + Testing + Context + Education + admitNIHSS + (1|participant); confidence interval (CI) calculated using the Wald method; *t*-tests calculated using Satterthwaite’s method.

**Table 4:** Summary of LASSO regression output.

Variable	Adjusted Coefficient	Coefficient	*z*-Score	*p*-Value	95% CI
Intercept	7.066 × 10^−17^	-	-	-	-
Age	−4.933 × 10^−2^	−0.305	−0.903	0.814	−0.832, 8.222
Education	−1.247 × 10^−1^	−0.510	−1.253	0.668	−1.216, 5.600
Hypoperfusion: ACA	−1.664 × 10^−1^	−0.215	−0.757	0.369	−2.319, 1.605
Hypoperfusion: MCA-parietal	−3.695 × 10^−1^	−0.664	−1.979	0.251	−1.298, 1.243
Angular gyrus	−3.073 × 10^−1^	−0.412	−1.299	0.262	−1.033, 0.871
Thalamus	−3.597 × 10^−1^	−0.538	−1.908	0.291	−1.068, 1.217
ILF	−5.827 × 10^−1^	−0.847	−2.306	0.379	−1.563, 2.642
IFOF	−5.120 × 10^−1^	−0.852	−2.403	0.210	−1.515, 1.255

ACA = anterior cerebral artery; MCA = middle cerebral artery; ILF = inferior longitudinal fasciculus; IFOF = inferior fronto-occipital fasciculus.

## Data Availability

Available upon request.

## Supplementary Materials

The following are available online at https://www.mdpi.com/article/10.3390/brainsci11050667/s1, Table S1: List of real-word sentences assessed during affective prosody recognition.
