# Supplementary material for: Explicit Training to Improve Affective Prosody Recognition in Adults with Acute Right Hemisphere Stroke
Source: Brain Sci. 2021 May 20;11(5):667. doi: 10.3390/brainsci11050667 (PMC8161405; doi:10.3390/brainsci11050667)
Supplement: Supplementary file 1 [file brainsci-11-00667-s001.zip › brainsci-1214704-supplementary.pdf]

**Supplementary Materials**

Table S1. List of real-word sentences assessed during affective prosody recognition.

| Emotion | Sentence |
|---|---|
| Happy | She went to the store to get more |
| Happy | The teacher wrote three names down |
| Happy | She did not know him before this |
| Happy | We walked through the park to get here |
| Sad | This is his first day at the office |
| Sad | She read the story in the newspaper |
| Sad | He sold his car to his neighbor |
| Sad | She ordered a cake for Thursday |
| Angry | Her husband came to the party |
| Angry | She cooked pancakes for breakfast |
| Angry | We bought shoes for the children |
| Angry | He ordered another pizza for us |
| Afraid | I never saw that person before today |
| Afraid | I will be going home today |
| Afraid | They bought a new boat |
| Afraid | We had hamburgers for supper |
| Surprised | The woman was running by our house |
| Surprised | She will be going to the new school |
| Surprised | They bought the house next door |
| Surprised | This is the second time she competed |
| Bored | The man knocked on our front door |
| Bored | He will talk to her about the book |
| Bored | He went to the other race |
| Bored | He paid the bill on Friday |

Note: Only *happy, sad, angry,* and *afraid* sentences were included in analyses.
